# Supplementary material for: Decrease in Myelin-Associated Lipids Precedes Neuronal Loss and Glial Activation in the CNS of the Sandhoff Mouse as Determined by Metabolomics
Source: Metabolites. 2020 Dec 30;11(1):18. doi: 10.3390/metabo11010018 (PMC7823728; doi:10.3390/metabo11010018)

Supplementary Data: Decrease in myelin-associated lipids precedes neuronal loss and glial activation in the CNS of the Sandhoff mouse as determined by metabolomics

Emmanuelle Lecommandeur [1], Maria Begoña Cachón-González [2], Susannah Boddie[1], Ben McNally[1], Andrew W. Nicholls [3], Timothy M. Cox [2] & Julian L. Griffin [1,4*]

[1] Department of Biochemistry and Cambridge Systems Biology Centre, University of Cambridge, Cambridge, CB2 1GA UK.

[2] Department of Medicine, Cambridge Biomedical Campus, Cambridge, CB2 0QQ, UK.

[3] GSK, Stevenage, SG1 2NY, UK.

[4] UK Dementia Research Institute at Imperial College; Burlington Danes Building, Imperial College London, Hammersmith Campus, Du Cane Road, London, W12 0NN; Section of Biomolecular Medicine, Division of Systems Medicine, Department of Metabolism, Digestion and Reproduction, The Sir Alexander Fleming Building, Imperial College London, Exhibition Road, South Kensington, London, SW7 2AZ, UK.

* Correspondence: jlg30@ic.ac.uk, +44 (0)20-7594 3220

Supplementary Figure 1: Concentration of GA2 (d18:1/18:0) and GA2 (d18:1/20:0) calculated as the average integrated area under the curve of the negative ion mode LC-MS chromatogram from the analysis of cerebrum tissue from control and *Hexb* $^{-/-}$ mice. A. Concentration of GA2 (d18:1/18:0). **B**. Concentration of GA2 (d18:1/20:0). n= 5 for each group. Results are mean + standard error of the mean (SEM). Significance level quoted for Student's *t*-test * $p \leq 0.05$; ** $p \leq 0.01$; *** $p \leq 0.001$.

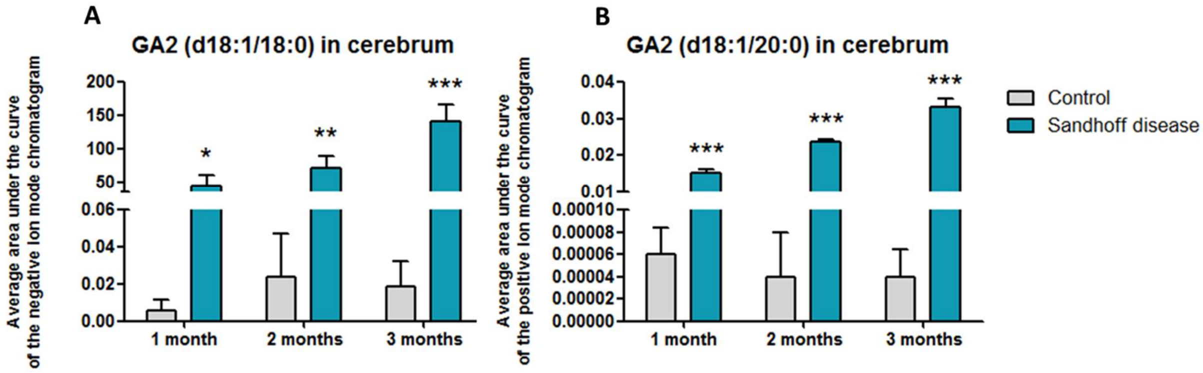

Supplement: Supplementary file 1 [file metabolites-11-00018-s001.pdf]
